# Nanoarhitectonics of Inorganic–Organic Silica–Benzil Composites: Synthesis, Nanocrystal Morphology and Micro-Raman Analysis

**DOI:** 10.3390/nano13131913

**Published:** 2023-06-23

**Authors:** Yaroslav Shchur, Andrii Bendak, Guillermo Beltramo, Anatoliy S. Andrushchak, Svetlana Vitusevich, Denys Pustovyj, Bouchta Sahraoui, Yurii Slyvka, Andriy V. Kityk

**Affiliations:** 1 Institute for Condensed Matter Physics, 1 Svientsitskii Str., 79011 Lviv, Ukraine; 2 Department of Applied Physics and Nanomaterials Science, Lviv Polytechnic National University, 12 S. Bandery Str., 79013 Lviv, Ukraine; 3 Institute of Biological Information Processing Mechanobiology (IBI-2), Forschungszentrum Juelich, D-52425 Juelich, Germany; 4 Institute of Bioelectronics (IBI-3), Forschungszentrum Juelich, D-52425 Juelich, Germany; 5 LPHIA, SFR MATRIX, University of Angers, 2 Bd. Lavoisier, CEDEX 01, 49045 Angers, France; 6 Department of Inorganic Chemistry, Ivan Franko National University of Lviv, Kyryla i Mefodiya Str., 6, 79005 Lviv, Ukraine; 7 Faculty of the Electrical Engineering, Czȩstochowa University of Technology, Al. Armii Krajowej 17, 42-200 Czȩstochowa, Poland; andriy.kityk@univie.ac.at

**Keywords:** Raman scattering, organic–inorganic nanocomposites, (C_6_H_5_CO)_2_, mesoporous SiO_2_, phonon, density functional theory, confinement effect

## Abstract

The synthesis of nanosized organic benzil (C${}_{6}$H${}_{5}$CO)${}_{2}$ crystals within the mesoporous SiO${}_{2}$ host matrix was investigated via X-ray diffraction, transmission electron microscopy, Raman spectroscopy, and *ab initio* lattice dynamics analysis. Combining these methods, we have proved that the main structural properties of benzil nanocrystals embedded into SiO${}_{2}$ host membranes with pore diameters of 6.0, 7.8, 9.4, and 13.0 nm are preserved compared to a bulk benzil crystal. Space confinement has an insignificant impact on the lattice vibrational properties of benzil crystals implanted into the host matrices. The *ab initio* lattice dynamics calculation of the phonon spectrum in the Brillouin zone center shows the mechanical and dynamical stability of benzil lattice, revealing very low optical frequency of 11 cm${}^{-1}$ at point Γ.

## 1. Introduction

The design of new functional materials consisting of two or more nanosized components has recently attracted great interest, as it opens up wide opportunities for creating materials with unique properties [1,2]. Special attention has been paid to nanoporous materials, which play the role of a host substance that can be filled with various guest compounds. There is a wide range of nanocomposite materials, i.e., multi-phase formations in which at least one of the phases has one, two, or three dimensions smaller than 100 nanometers. Guest nano-objects of various morphologies, including carbon nanotubes [3,4,5], graphene nanoplates [6], nanowires [7,8,9], nanoparticles and nanospheres [10,11,12], quantum dots [13], and nanocrystals [14,15,16], may be embedded into a composite host matrix, such asa polymer, gel, or nanoporous crystalline medium. Often, the filling materials bring about the main functional properties of the newly created substance, which are not characteristic of the unfilled host matrices. However, in many cases, one needs to take into account the properties and morphology of the host medium, which can significantly affect the final desired characteristics of a composite material.

Multifunctional nanocomposite materials are widely used in various spheres of human innovative activity, such as information storage, energy harvesting applications, spintronics, bioengineering applications, medicine, drug delivery, cosmetics, etc. [3,10,13].

Recently, we investigated the formation of KH${}_{2}$PO${}_{4}$ and Ba(NO${}_{3}$)${}_{2}$ nanoscale crystals in a mesoporous host SiO${}_{2}$ matrix [14,15] and found that, to a great extent, they resemble the structure and lattice vibration properties of their bulk counterparts. In this paper, we report the creation of a newly synthesized nanocomposite material, SiO${}_{2}$:(C${}_{6}$H${}_{5}$CO)${}_{2}$, based on a host mesoporous SiO${}_{2}$ matrix with tubular nanochannels of ∼6 to ∼13 nm in diameter filled with benzil (C${}_{6}$H${}_{5}$CO)${}_{2}$ nanocrystals. Benzil is often referred to as “organic quartz” due to having the same symmetry as α-quartz and similar physical properties, e.g., optical activity, piezoelectricity, linear electro-optics (Pockels effect), and second-order optical nonlinearity (second harmonic generation (SHG) effect). Our research aims to solve the two-fold task.

Firstly, we are interested in the comparatively simple synthesis of composite material which would manifest the main physical properties of the bulk benzil crystal as a non-centrosymmetric crystal material. Our very recent study, in particular, shows that a silica–benzil nanocomposite can be considered as an efficient nonlinear optical (NLO) material for light–frequency conversion based on three-wave mixing processes, wherein spatial confinement reveals a significant impact on their conversion efficiency [17]. The SHG effect vanishes when spatial cylindrical confinement approaches the diameters of a few molecular layers, suggesting that the embedded benzil nanoclusters are not uniformly crystalline but are rather characterized by a more complex morphology consisting of an NLO-inactive disordered amorphous shell next to the channel wall and a well-ordered crystalline core participating in the NLO conversion. This result also clearly points to host–guest interfacial interactions, the role of which strongly increases under extreme spatial confinement.

Secondly, the problem of crystallization in ∼6 nm diameter pores of a complex-molecular-structure crystal such as benzil is not trivial. Owing to the comparative weakness of intermolecular interactions in this molecular crystal, one may not predict *a priori* the symmetry and structural peculiarities of nanocrystals grown in confined spatial circumstances. To observe the impact of space confinement on the benzil crystal structure, we prepared a series of silica–benzil SiO${}_{2}$:(C${}_{6}$H${}_{5}$CO)${}_{2}$ composites employing mesoporous SiO${}_{2}$ matrices with different pore diameters *D* ranging from ∼6 to ∼13 nm. The capillary penetration of molten benzil into the SiO${}_{2}$ substrate and its subsequent crystallization during cooling lead to the formation of crystalline benzil nanoclusters embedded in SiO${}_{2}$ nanochannels. Raman scattering and X-ray diffraction techniques aim to prove the formation of benzil nanocrystals inside nanochannels, as well as to explore the crystallographic texture and cluster morphology of synthesized silica–benzil nanocomposites. Raman micro-spectroscopy, on the other hand, is used here to characterize the microscale morphology of SiO${}_{2}$:(C${}_{6}$H${}_{5}$CO)${}_{2}$ composites, which turned out to be largely spatially inhomogeneous. Some lattice dynamical peculiarities of benzil were explained based on first-principles lattice dynamics calculations.

## 2. Experimental Section

The porous SiO${}_{2}$ templates, *p*SiO${}_{2}$, were prepared via the thermal oxidation (12 h, *T* = 1073 K) of the flow-through porous silicon membranes obtained through the electrochemical etching of highly *p*-doped Si(100) wafers (resistivity 0.01÷0.02 ohm·cm) using an electrolyte mixture of HF:C${}_{2}$H${}_{5}$OH (2:3) and *dc* current density of 12 mA/cm${}^{2}$. The etching process defines the morphology of porous silicon, which is characterized by nanochannels aligned along the [100] crystallographic direction, i.e., perpendicular to the wafer surface. The relevant tubular structure also remains in its oxidized counterpart, *p*SiO${}_{2}$ membranes. Their specific morphological characteristics such as porosity, *P*; channel diameter, *D*; and channel length (membrane thickness), *h*, depend on the etching time. Particularly, by varying the time from 2 to 8 h, we fabricated a series of *p*SiO${}_{2}$ membranes, hereafter referred to as *p*SiO${}_{2}$(6.0) [*D* = 6.0 ± 0.4 nm, *P* = 12%, *h* = 100 μm], *p*SiO${}_{2}$(7.8) [*D* = 7.8 ± 0.6 nm (*P* = 21%, *h* = 160 μm], *p*SiO${}_{2}$(9.4) [*D* = 9.4 ± 0.8 nm (*P* = 30%, *h*= 240 μm], and *p*SiO${}_{2}$(13.0) [*D* = 13.0 ± 1.0 nm (*P* = 49%, *h* = 310 μm] templates, respectively. The average channel diameters and the porosity were specified by analyzing the volumetric N${}_{2}$-sorption isotherms recorded at *T* = 77 K.

The electrochemical etching of silicon was found to cause depth-dependent pore widening, leading to a slightly conical shape of the nanochannels [18]. This feature is apparently also preserved in mesoporous silica. The nitrogen sorption experiments support this conclusion, since the samples with increasing thickness and correspondingly larger average channel diameters evidently reveal the broader pore size distribution due to longer electrochemical exposure (see Figure S1 in Supplementary Material). The analysis of volumetric N${}_{2}$-sorption isotherms provides only a rough evaluation. The inhomogeneity of the channel diameter along its long axis should not exceed 10–20%, while thicker *p*SiO${}_{2}$ membranes reveal the larger conicity of the channels.

Benzil nanocrystals were embedded into silica nanochannels via capillary crystallization. Mesoporous membranes were repeatedly washed with boiling acetone and ethanol and then dried at about 423 K for 1 h to remove the absorbed residues from the channel walls. The dried tubular *p*SiO${}_{2}$ matrices were then completely filled with a benzil melt via spontaneous capillary inhibition at a temperature a little above their melting point ($T>T_{m}$ = 368 K). A further cooling (2 K/min) down to room temperature led to the crystallization of the filler, i.e., the formation of benzil nanocrystals inside the silica nanochannels (see Figure S2 in Supplementary Material).

**Figure 1:**
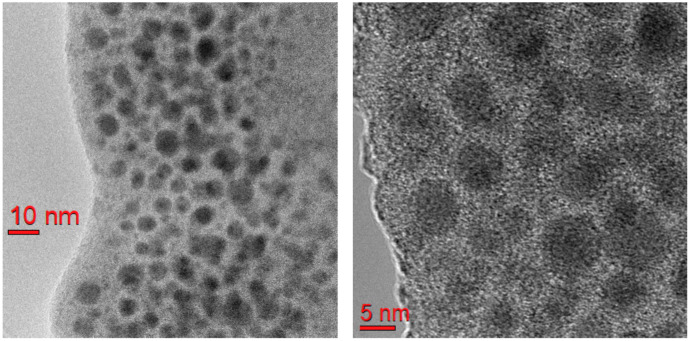
TEM images of SiO${}_{2}$:(C${}_{6}$H${}_{5}$CO)${}_{2}$ nanocomposite with a mean pore diameter of ∼6 nm.

The porous morphology of the SiO${}_{2}$:(C${}_{6}$H${}_{5}$CO)${}_{2}$ nanocomposite was visualized via the FEI Tecnai G${}^{2}$ F20 high-resolution transmission electron microscopy (HRTEM) with 200 kV acceleration voltage and 0.19 nm point resolution. The sample for the HRTEM study was prepared as a thin (∼87 nm) lamella with a surface of 10 × 20 μm${}^{2}$. The TEM image of the SiO${}_{2}$:(C${}_{6}$H${}_{5}$CO)${}_{2}$ nanocomposite with the smallest pore diameter of 6 nm is depicted in Figure 1. As seen in this figure, there is a set of randomly distributed pores of SiO${}_{2}$ host matrix with a mean pore diameter of ∼6 nm.

**Figure 2:**
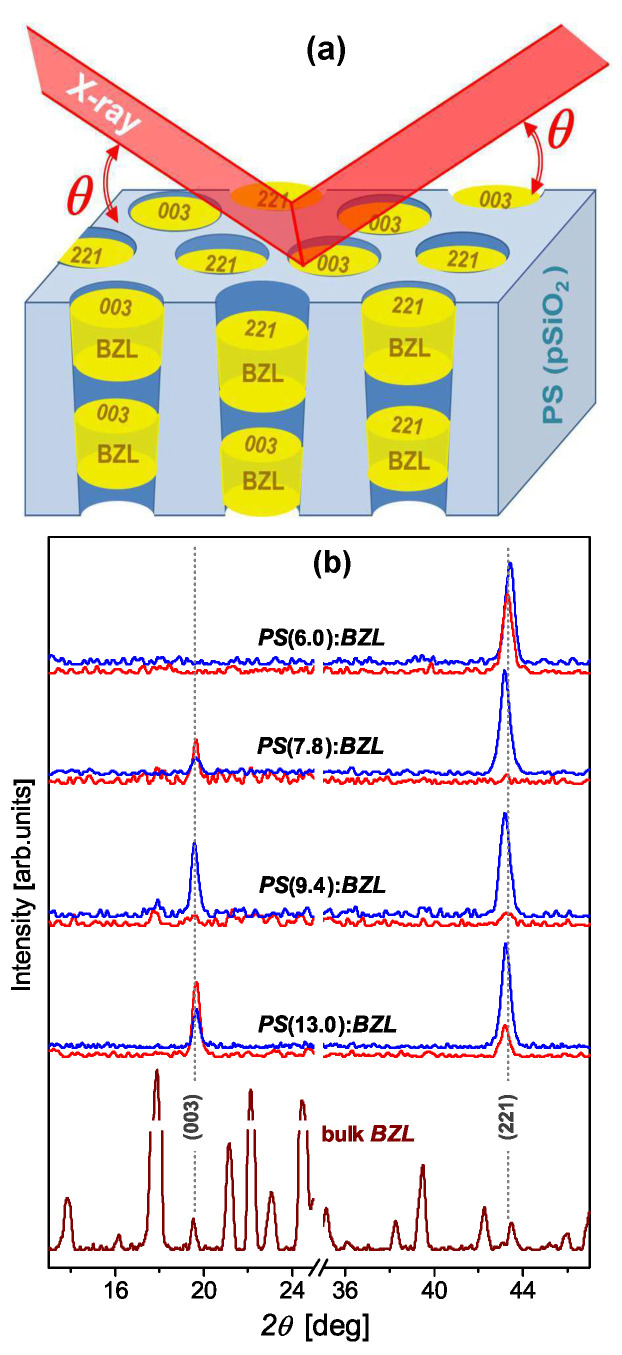
(**a**) Sketch of silica–benzil (*p*SiO${}_{2}$:benzil) nanocomposite structure. Slow cooling through the crystallization point ($T_{m}$ = 369 K) leads to the formation of texture consisting of benzil (BZL) crystalline nanoclusters inside the silica channels. (**b**) Background-subtracted XRD patterns (θ/2θ-scan) recorded from the opposite sides (red and blue line colors) of nanocomposite *p*SiO${}_{2}$(*D*):benzil (PS(*D*):BZL) with different channel diameters (D= 6.0, 7.8, 9.4, and 13.0 nm). The reference powder XRD pattern of bulk benzil is shown for comparison. Diffraction Bragg geometry is described in panel (**a**) of this figure.

Capillary crystallization in nanochannels is accompanied with a strong opalescence scattering apparently caused by the appearance of solidified capillary bridges, i.e., benzil crystalline nanoclusters separated by voids as a result of the filler volume contraction during its crystallization (see Figure 2a). The experimental study of crystallization processes in nanocomposite materials is quite challenging. The X-ray diffraction (XRD) technique (DRON-4 diffractometer, CuK${}_{\alpha }$ radiation) was used here to explore the formation of the preferred oriented crystals in the pore space, aiming to characterize the textural morphology of the synthesized inorganic–organic silica–benzil nanocomposites. We relied on diffraction Bragg geometry, as described in Figure 2a. For this method, the atomic planes perpendicular to the long pore axis are probed. Figure 2b displays the background-subtracted XRD diffractograms recorded for *p*SiO${}_{2}$:benzil nanocomposites with different channel diameters (D= 6.0, 7.8, 9.4, and 13.0 nm). The powder XRD pattern of the bulk benzil serves here as a reference for comparison. Even a simple analysis of the nanocomposite XRD patterns, in particular their comparison with the reference diffractogram of the bulk benzil, clearly shows that the orientations of the nanocrystalline clusters are not random. The observed Bragg reflections reveal benzil nanoclusters of two crystallographic orientations, (221) and (003), wherein their ratio is confinement dependent. On the contrary, at small channel diameters, the textural morphology is dominated by crystalline (221) nanoclusters; at larger channel diameters, fractions of (221) and (003) nanoclusters tend towards parity. Surprisingly, the intensities of the relevant Bragg reflections recorded from the opposite sides of the nanocomposite membranes distinctly differ from each other (see Figure 2b, red and blue lines), suggesting that the benzil texture is orientation and density inhomogeneous along the nanochannels, which is also confirmed by the Raman microscopy measurements presented below. This feature of silica-based nanocomposites, which was also revealed in previous studies [14,15], should presumably be attributed, among other features, to the slightly conical shape of the nanochannels. This causes the formation of a capillary pressure gradient that forces the filler material to move along the nanochannels during its capillary crystallization. One may assume that the capillary crystallization takes place individually in spatially separated capillary bridges, wherein the formation of nanoclusters of different crystallographic orientations is a matter of local nonequilibrium thermodynamic conditions, specific interfacial host–guest interactions, and fluctuation effects under spatial confinement. By extracting the width of the Bragg reflection peaks and applying the Scherrer equation [19], one obtains practically the same average length, about 19–20 nm, for benzil nanoclusters along the long channel axis.

Needle-like polycrystalline benzil samples were of typical size; 15 × 0.2 × 0.2 mm${}^{3}$. Micro-Raman confocal microscopy measurements were carried out at room temperature in backscattering geometry using two Raman spectrometers, a Witec 300 alpha R setup with a spectral resolution of ∼1.0 cm${}^{-1}$, and a Renishaw inVia Reflex spectrometer with a spectral resolution of ∼1.6 cm${}^{-1}$. The 532 nm laser was utilized as a source of radiation. We used a Zeiss LD EC Epiplan-Neofluar ×50/0.55 objective (Witec) and Leica (DMI 2700) microscope (Renishaw). To prevent the sample melting, a laser spot on the sample surface was defocused by 70% with the laser power ≤5 mW. An edge filter was used to separate the Raman response from the excitation line (Witec). We utilized a Newton Andor EMCCD camera with 1600 × 200 pixels as a detector (for Witec set-up) and a Renishaw Centrus CCD detector with 1024 × 256 pixels (for Renishaw set-up). Owing to the setup limitations, our Raman spectra were recorded only above 100 cm${}^{-1}$.

The calculations of the vibrational spectrum were carried at the Brillouin zone center (point Γ) within the density functional perturbation theory (DFPT) using generalized gradient approximation (GGA) in Perdew–Burke–Ernzerhof parameterization [20]. The long-range dispersion corrections published by Grimme et al. [21] (GGA+DFT-D3 approach) were also taken into account. The *ab initio* calculations were performed using the package ABINIT [22,23]. The norm-conserving ONCVPSP pseudopotentials were utilized with O(2s${}^{2}$2p${}^{4}$), C(2s${}^{2}$2p${}^{2}$), and H(1s${}^{1}$) valence states. The convergence analysis was carried out concerning both the Brillouin zone sampling using the Monkhorst-Pack scheme [24] and the kinetic energy cut-off for plane–wave calculations. The experimental crystal structure detected at room temperature [25] was relaxed during the theoretical structural optimization, which was performed within the Broyden–Fletcher–Goldfarb–Shanno scheme [26]. The optimized calculated lattice parameters a = 8.418 and c = 13.417 *Å* deviated from the corresponding experimental data [25] by 0.5 and 2.1%, respectively. The maximum forces acting on each atom were lower than 5.9 × 10${}^{-9}$ eV/*Å*. The 5 × 5 × 5 grid for Brillouin zone sampling and the cut-off energy E${}_{cut}$ = 1224 eV with a cut-off smearing of 13.6 eV were used.

## 3. Results and Discussion

### 3.1. Symmetry Considerations

**Figure 3:**
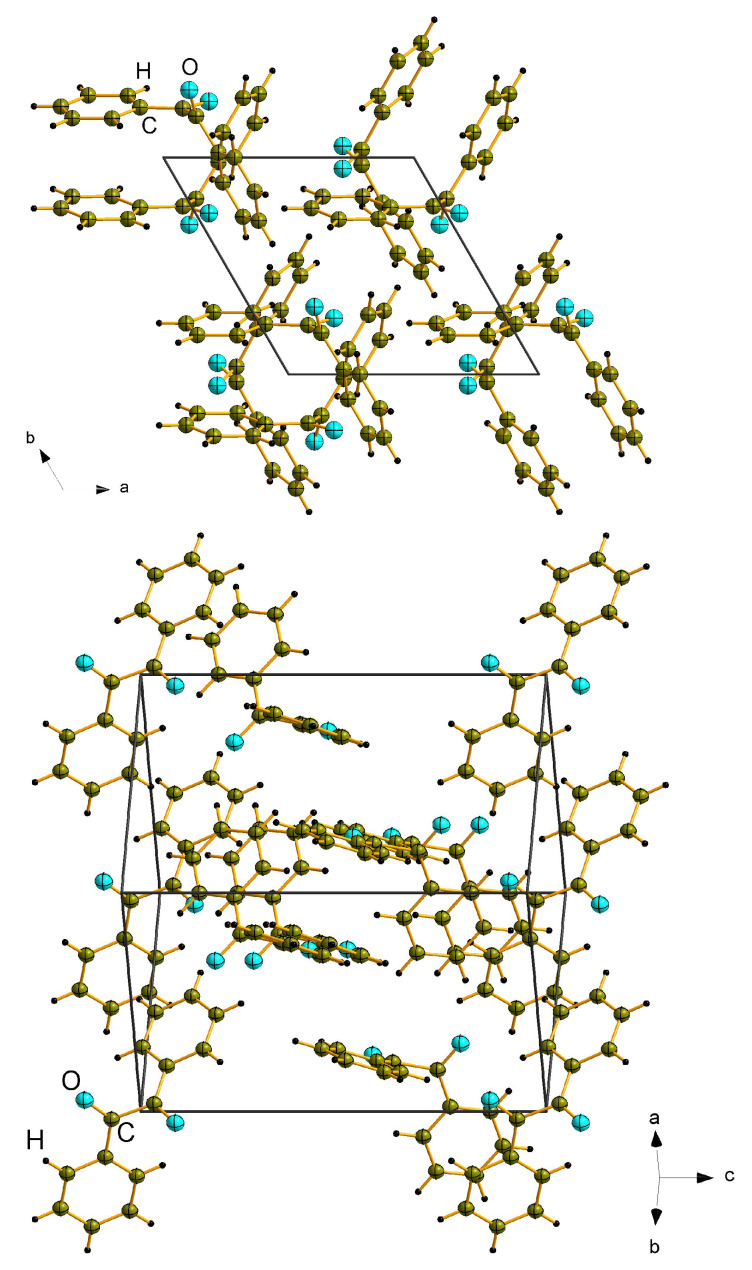
Crystal structure of benzil at room temperature [25] (space group *P3${}_{1}$21*, No. 152).

At room temperature, benzil adopts a trigonal structure with space group *P3${}_{1}$21* (No. 152) or enantiomorphic *P3${}_{2}$21* (No. 154) space group. The unit cell contains three structural units (Z = 3) ordered spirally along the *c* axis (see Figure 3). At 83.5 K, benzil undergoes a ferroelectric/ferroelastic structural phase transition to a monoclinic phase (*P2${}_{1}$*, sp. gr. No. 4), which is accompanied with the unit cell multiplication (Z = 6) [27,28].

The room-temperature vibrational spectrum is very rich. A total of 234 phonon modes were classified according to the irreducible representations (irreps) of the *P3${}_{2}$21* group at point Γ as follows,

$$\Gamma (234\;\mathrm{modes})\;=\;39A{}_{1}\left(R\right)\;+\;39A{}_{2}\left(\mathrm{IR}\right)\;+\;78E(R,\mathrm{IR})$$

where R and IR imply the Raman or IR activity of the corresponding irreps.

Three benzil molecules placed in a unit cell and treated as rigid units may perform nine translational (three of which are acoustic) and nine rotational vibrations. The rest of the vibrations are internal ones comprising all possible movements of the phenyl rings of C${}_{6}$H${}_{5}$ as the whole constituents; deformations of the phenyl rings; and C-C, C=O, and H-C vibrations within the same molecule. Following the general group theory consideration of paper [29], one may perform the normal-mode classification as follows:

$$\Gamma_{external}\left(18\right)\;=\;2A{}_{1}\;+\;4A{}_{2}\;+\;6E$$

$$\Gamma_{acoustic}\left(3\right)\;=\;A{}_{2}\;+\;E$$

$$\Gamma_{translational}\left(6\right)\;=\;A{}_{1}\;+\;A{}_{2}\;+\;2E$$

$$\Gamma_{rotational}\left(9\right)\;=\;A{}_{1}\;+\;2A{}_{2}\;+\;3E$$

$$\Gamma_{internal}\left(216\right)\;=\;37A{}_{1}\;+\;35A{}_{2}\;+\;72E.$$

The Raman spectrum should contain as many as 116 optic modes, 77 of which are two-fold degenerate owing to the two-dimensionality of E irrep.

### 3.2. Ab Initio Calculations

To check the mechanical stability of benzil crystals in the *P3${}_{2}$21* phase, one needs to calculate the whole matrix of elastic moduli C${}_{ij}$. The comparison between the calculated C${}_{ij}$ components and those detected experimentally by means of Brillouin scattering [30] is presented in Table 1. As seen in this table, the calculated values of C${}_{ij}$ are somewhat overestimated compared to their experimental counterparts. However, the general tendency in their relative values, except for C${}_{44}$, is well preserved. In general, the elastic moduli C${}_{ij}$ of benzil are several times smaller than those of a quartz crystal [31], which indicates the low mechanical stability of the benzil compound. Having calculated the elastic moduli, one may state that the Born stability criteria [32,33] for unstressed trigonal crystal

$$C{}_{11}>|C{}_{12}|,\;C{}_{44}>0,\;C{}_{13}^{2}<\frac{1}{2}C{}_{33}(C{}_{11}+C{}_{12}),\;C{}_{14}^{2}<\frac{1}{2}C{}_{44}(C{}_{11}-C{}_{12})$$

are nicely satisfied.

Benzil belongs to the piezoelectric *32* crystal class, which admits two independent piezoelectric coefficients, e${}_{11}$ and e${}_{14}$. It is instructive to compare the values e${}_{11}$ = 76.1 and e${}_{14}$ = −26.9 mC/m${}^{2}$, calculated by us with those experimentally measured in “canonical” piezoelectric crystal of the same *32* symmetry, α-quartz, i.e., e${}_{11}$ = 171.0 and e${}_{14}$ = −40.6 mC/m${}^{2}$ [31]. The calculated e${}_{11}$ and e${}_{14}$ values are about two times smaller than the corresponding values of quartz crystals.

The numerical calculation of vibrational frequencies of an isolated benzil molecule was formerly published in a few papers [35,36]. However, in these cases, only 72 vibrational modes of an isolated molecule were taken into account (26 atoms × 3 = 78—three acoustic and three rotational modes). The lattice dynamics treatment of benzil crystal takes into consideration the whole set of 234 normal modes inherent for three benzil molecules. To the best of our knowledge, the present study is the first numerical lattice dynamics study of a benzil crystal which takes into consideration the intermolecular vibrations.

The phonon frequencies calculated in the Brillouin zone center and classified according to the irreps of the *P3${}_{1}$21* space group are listed in Table 2. In this table, we also present the experimental data detected by us in the unpolarized Raman spectra on the powdered benzil sample. There is reasonable correspondence between the calculated frequencies and those observed in our Raman scattering experiment. Since there are no imaginary frequencies in the calculated spectrum, one may state that the benzil vibrational spectrum reveals a dynamic stability at point Γ. We do not intend to analyze the entire vibrational spectrum of benzil crystal in detail since this is beyond the main purpose of our research and, to a large extent, this was undertaken in [35,36]. We focus on the low-frequency part of the phonon spectrum which contains the modes responsible for structural phase transitions, which were the subject of discussions of the formerly published papers [27,37,38,39].

Based on the analysis of the calculated *eigen* vectors, one may infer that all purely external vibrations are located below ∼70 cm${}^{-1}$. All modes of frequencies higher than 70 cm${}^{-1}$ are of very complicated mixed types which involve vibrations of all structural units of benzil crystals. Owing to the apparatus limitations of our Raman scattering spectrometers, we were able to measure the Raman spectra above 100 cm${}^{-1}$. In order to check the reliability of our calculations in the low-frequency part of the spectrum below 100 cm${}^{-1}$, we list in Table 2 the experimental data of paper [37]. According to the symmetry conditions, seven external phonon modes, 2A${}_{1}$ + 5E, should be visible in the Raman spectrum. Instead of two, three low-frequency lines of A${}_{1}$ type were recorded in the Raman spectrum below 70 cm${}^{-1}$, namely, ∼29, 39, and 69 cm${}^{-1}$ [37,38]. To match the experimental results with the factor group requirements, the authors of papers [37,38] assumed that the lowest-energy mode near 29 cm${}^{-1}$ is a difference tone between some higher-frequency modes, i.e., 39 and 69 cm${}^{-1}$. According to our calculations, the first low-frequency A${}_{1}$ mode is located near 28 cm${}^{-1}$ and should be treated as intrinsic A${}_{1}$ phonon mode. The 69 cm${}^{-1}$ experimental mode of the A${}_{1}$ species should not be treated as the external vibrational mode since the nearest calculated A${}_{1}$ mode at 73.6 cm${}^{-1}$ is of a mixed external–internal type mode. The similar criteria should be applicable to the modes of E symmetry. According to our *eigen* vector analysis, only four lowest-frequency E-type modes, 11.0, 39.2, 45.6, and 63.8 cm${}^{-1}$, are pure external lattice modes instead of the five modes admitted by the group theory analysis. The fifth calculated E-mode near 76.9 cm${}^{-1}$ is of mixed external–internal type.

As seen in Table 2, the lowest calculated frequency at 11.0 cm${}^{-1}$ corresponds to E irrep. This doubly degenerated optic mode has an experimental counterpart at 16 cm${}^{-1}$, observed in the Raman spectrum [37], which manifests a significant temperature softening while approaching the phase transition point at 83.5 K [38]. However, despite the large temperature softening of this mode near point Γ, the structural phase transition is evidently evoked by the softening of the same lowest-energy phonon branch near point M (1/2, 0, 0), leading to the unit cell doubling in the low-temperature phase. This phonon temperature evolution was experimentally proved by inelastic neutron scattering in the deuterated analog (C${}_{6}$D${}_{5}$CO)${}_{2}$. It turned out that lowest-frequency branch softens with temperature at the M-point faster than the Γ-point mode [39]. However, the real mechanism of structural phase transition in benzil appears to be more intricate due to the significant impact of very complex network of hydrogen bonds in this crystal. Based on the diffuse neutron scattering, the authors of paper [28] showed that the intramolecular vibrational mode at ∼8.95 cm${}^{-1}$ in benzil crystals is coupled with the shearing motion of the hydrogen-bonded network linking the neighboring molecules. It is worth noting that even the much simpler network of hydrogen bonds of KH${}_{2}$PO${}_{4}$-type crystals plays a definitive role in structural-phase transitions, see, e.g., [40,41,42,43,44].

According to our calculations, the transverse–longitudinal (TO-LO) splitting of polar modes inherent for the polar vibrations of A${}_{2}$ (dipole moment along *z*-axis) and E (dipole moment lies in (*x*, *y*) plane) symmetry is incredibly small, of the order of a few tenths of cm${}^{-1}$ or even less. Only 10 optic modes out of 38 modes of A${}_{2}$ symmetry reveal TO-LO splitting lager than 1 cm${}^{-1}$, normally between 1 and 2 cm${}^{-1}$. The similar values of TO-LO splitting are observable for the calculated normal modes of the E species, where only 8 optic modes out of 77 show TO-LO splitting of 1 to 2 cm${}^{-1}$, and only two modes at 1212.7 and 1642.3 cm${}^{-1}$ reveal TO-LO splitting near 5 cm${}^{-1}$. Such a small TO-LO splitting implies the insignificance of the macroscopic electric field that creates such a split in dielectric crystals. Note that the weak TO-LO splitting of the low-frequency modes of E symmetry was formerly detected in the Raman spectra [38].

### 3.3. Raman Scattering

**Figure 4:**
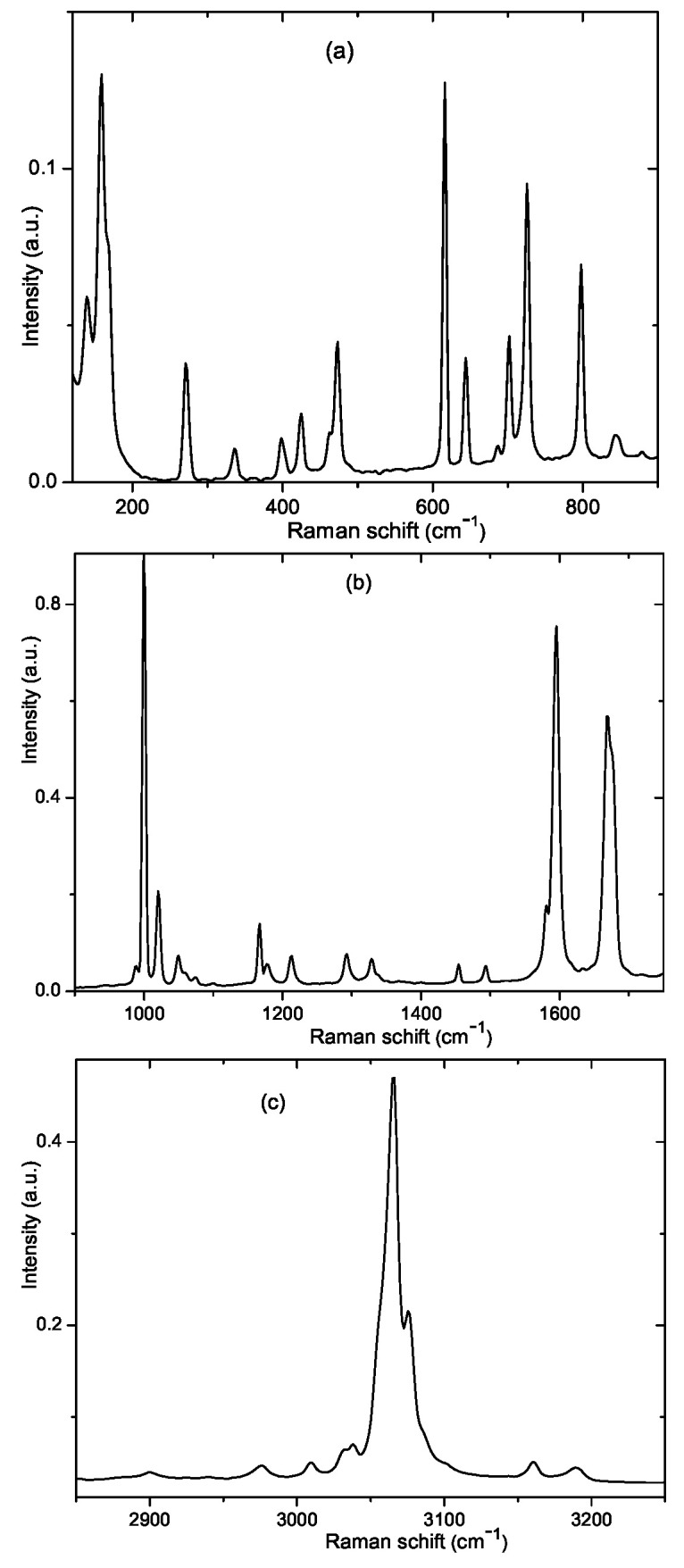
Raman spectrum of benzil single crystal at room temperature presented in different frequency ranges, (**a**)—120–900, (**b**)—900–1750 and (**c**)—2850–3250 cm${}^{-1}$.

The unpolarized Raman spectrum taken from the benzil powdered sample at room temperature is depicted in Figure 4. We observed far fewer phonon vibrations than those theoretically admitted by lattice dynamics for benzil crystals. Instead of the 116 optic modes theoretically acceptable for observation in the Raman spectrum, we detected about 50 modes above 100 cm${}^{-1}$. Owing to the large mass of the benzil molecules and to their weak intermolecular interactions inherent for this molecular crystal, external phonon modes were characterized by very low frequencies, which were below the lower wave number limit of our experimental setup (100 cm${}^{-1}$).

For a better resolution, we divided the Raman spectrum into three different parts (Figure 4). However, the physical meaning of such a separation is valid only for the highest frequency range, near 3080 cm${}^{-1}$, which corresponds to C-H vibrations. The spectral region between 100 and ∼1700 cm${}^{-1}$ contains internal vibrations of phenyl rings and vibrations of C-C and C=O groups.

Raman frequencies experimentally observed in the bulk benzil crystal are listed in Table 2. It should be noted that we detected practically identical Raman spectra with an accuracy of ±1.0 cm${}^{-1}$ using Witec and Renishaw spectrometers. Our Raman spectra were measured in unpolarized light. Therefore, choosing the experimental phonon frequencies for comparison with the calculated ones, we imposed no symmetry restrictions, but only set the requirements for the closest agreement between calculations and experiment.

In general, the Raman spectrum from SiO${}_{2}$:(C${}_{6}$H${}_{5}$CO)${}_{2}$ nanocomposites contains constituents of both nanoporous amorphous SiO${}_{2}$ membranes (see Figure S3 in Supplementary Material) and nanosized benzil crystals embedded in the host matrix. Note that the Raman spectra from pure SiO${}_{2}$ matrices with different pore diameters are basically the same. Since the SiO${}_{2}$ Raman spectrum has a much lower intensity than that of the SiO${}_{2}$:(C${}_{6}$H${}_{5}$CO)${}_{2}$ nanocomposite, we observed no clear peaks from the nanoporous, amorphous SiO_2_ membrane in the total SiO${}_{2}$:(C${}_{6}$H${}_{5}$CO)${}_{2}$ spectrum. Even the most intensive broad band of the SiO${}_{2}$ spectrum, centered near 430 cm${}^{-1}$ with full widths at half maximum (FWHM) 200 cm${}^{-1}$, was not detected in the Raman spectrum of the nanocomposite.

**Figure 5:**
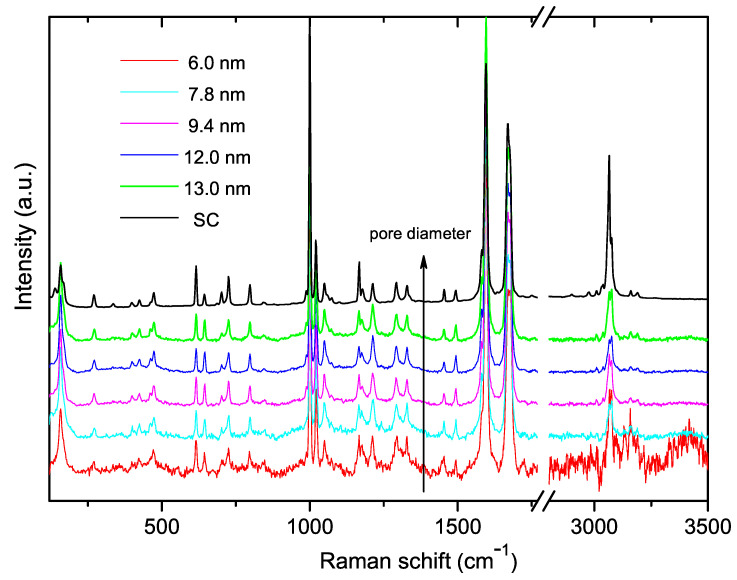
Raman spectra taken from SiO${}_{2}$:(C${}_{6}$H${}_{5}$CO)${}_{2}$ nanocomposites with different pore diameters, ranging from 6.0 to 13.0 nm, and from benzil bulk crystal. The arrow indicates the Raman spectra taken from matrices with increasing pore diameter.

The comparison of the Raman spectra taken from the compounds newly synthesized in host SiO${}_{2}$ matrices of various pore diameters, 6.0, 7.8, 9.4, 12.0, and 13.0 nm, and that were recorded from the bulk powdered (C${}_{6}$H${}_{5}$CO)${}_{2}$ benzil crystal, is depicted in Figure 5. As seen in this figure, there is an impressive similarity between all presented spectra. We scrupulously investigated the normalized intensity and FWHM of all lines in various frequency regions of the Raman spectra presented in Figure 5 and found no noticeable difference between them. The larger scattering noise of the Raman spectrum observed on the plate with the smallest 6.0 nm diameter may be caused by the smallest amount of the filling benzil compound due to the smallest 12 % porosity of this plate. Raman spectra were measured in different places of the SiO${}_{2}$:(C${}_{6}$H${}_{5}$CO)${}_{2}$ plates and from both their sides. Moreover, in order to check the uniformity of filling the guest benzil compound into pores of the host SiO${}_{2}$ matrices, we measured the spectra from the freshly broken membrane edges. Figure 5 contains exactly the spectra measured at the broken edges of all analyzed nanocomposites.

**Figure 6:**
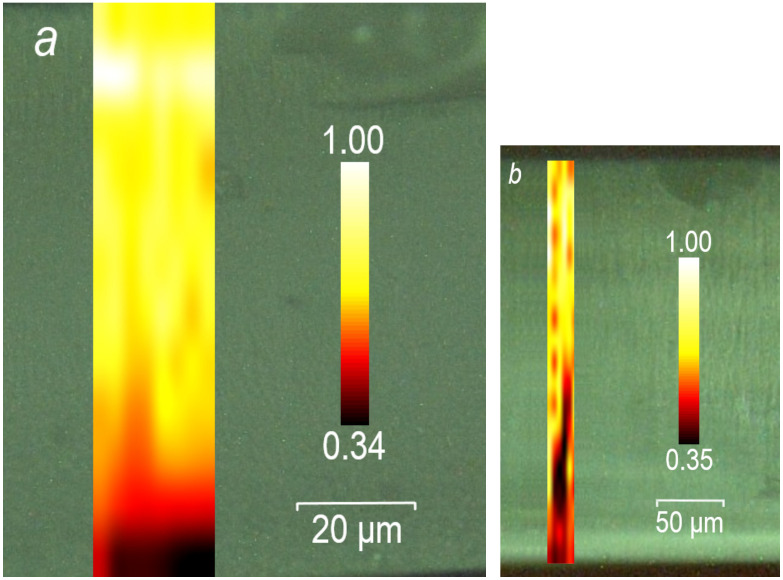
Raman mapping of the freshly broken edge of nanocomposite SiO${}_{2}$:(C${}_{6}$H${}_{5}$CO)${}_{2}$ membranes. (**a**) Pore diameter is 6 nm, mapping area is 21 × 110 μm${}^{2}$, 8 × 12 digits, (**b**) pore diameter is 13 nm, mapping area 20 × 300 μm${}^{2}$, 6 × 19 digits. Normalized intensity of Raman line near 1000 cm${}^{-1}$ decreases almost uniformly from the top to the bottom of the plate.

The Raman mapping of the freshly broken edges of the two SiO${}_{2}$:(C${}_{6}$H${}_{5}$CO)${}_{2}$ membranes with pore diameters of 6 nm, Figure 6a and 13 nm Figure 6b, through the whole thickness of the plates within the area sized 21 × 110 and 20 × 300 μm${}^{2}$, respectively, is depicted in Figure 6. Here, we present the results from the membranes with two marginal pore diameters, 6 and 13 nm. The membranes with all intermediate pore diameters manifest a similar result. A very intensive line near 1000 cm${}^{-1}$ was chosen for this mapping. As seen in this figure, the intensity of this line almost uniformly decreases from the top to the bottom of the sample. This decrease in the Raman line intensity may be caused by a decrease in the pore diameter (conical shape) moving from one side of the plate to the other one due to the peculiarity of the electrochemical etching during the creation of the SiO${}_{2}$ membranes.

Similar Raman mapping was performed on two surfaces of all the SiO${}_{2}$:(C${}_{6}$H${}_{5}$CO)${}_{2}$ plates (not shown). We observed a nearly homogeneous distribution of Raman line intensities over the numerous scanned areas, which testifies to the homogeneous arrangement of benzil crystal over the surfaces of the membranes.

A careful inspection of the mode frequencies, line shapes, and their intensities of the Raman spectra recorded from the SiO${}_{2}$:(C${}_{6}$H${}_{5}$CO)${}_{2}$ membranes with all available pore diameters 6.0, 7.8, 9.4, and 13.0 nm (see Figure 5), indicates a very close resemblance of all the presented spectra with the spectrum of the bulk benzil crystal. With great confidence, one may state that the spectroscopic data confirm the crystallization of benzil nanocrystals inside the pore network of all SiO${}_{2}$ matrices used in our research. Neither benzil crystal nor benzil molecular structure are not distorted by the space confinement effect. This is a rather unexpected result, considering our previous experimental data concerning the impact of the nanoconfinement effect on the Raman spectra of KH${}_{2}$PO${}_{4}$ [14] and Ba(NO${}_{3}$)${}_{2}$ [15] nanocrystals embedded into SiO${}_{2}$ matrices. However, the lack of a distinct impact of the space confinement effect on the vibrational spectrum of benzil may be explained by two reasons. The first reason may be related to the restriction of our experimentally available, low-frequency limit of ∼100 cm${}^{-1}$. As was already shown above based on our *ab initio* eigen-vector analysis, the external lattice vibrations were placed below ∼70 cm${}^{-1}$. Exactly this low-frequency part of the phonon spectrum provides information about the general crystal symmetry of a compound, and any distortion of the crystal structure should be mainly visible in this low-frequency region. The second reason is a very complex and dense network of hydrogen bonds in the benzil crystal. A large number of weak hydrogen bonds stabilizes the intra- and intermolecular structure of benzil nanocrystals embedded in nanosized channels of the SiO${}_{2}$ matrix, preventing their spatial distortion.

## 4. Conclusions

We performed a detailed spectroscopic analysis of an artificial material composed of a nanoporous SiO${}_{2}$ matrix with pores of various diameters, from 6 to 13 nm, filled with benzil nanocrystals. Using the Raman mapping of different places on the membrane surface and freshly broken edges, we proved that the Raman responses of SiO${}_{2}$:(C${}_{6}$H${}_{5}$CO)${}_{2}$ nanocomposites are almost identical to those of bulk benzil crystals. XRD measurements performed on the samples with different pore diameters proved that the pores of the SiO${}_{2}$ matrices are preferably dominated by benzil nanocrystals of (221) and (003) crystallographic orientations. The nanocrystals of the (221) orientation are mostly typical of the pores of the smallest, 6 nm, diameter. The density of the nanocrystals with the other (003) orientation increases in SiO${}_{2}$:(C${}_{6}$H${}_{5}$CO)${}_{2}$ nanocomposites with the increasing pore diameter from 6 to 13 nm. The HRTEM experiment proved the random distribution of the pore diameters.

The phonon spectrum calculated in the Brillouin zone center within the first-principles approach revealed both dynamic and mechanic lattice stability conditions. Spectroscopic data were interpreted based on the results of the lattice dynamics simulations.

We found no spectroscopic evidence of the impact of the space confinement effect on the crystal and molecular structure of nanosized benzil crystals compared with bulk benzil crystals in pores of the smallest, 6 nm, diameter. The lack of spatial distortions of nanosized benzil crystals may be related either to their low-frequency manifestation, which is not detectable due to the experimental limitation of our setup, or due to the impact of the dense hydrogen bond network, which stabilizes the benzil crystal structure even in spatially confined conditions.

## Figures and Tables

**Table 1:** Elastic moduli C${}_{ij}$ (in GPa) and piezoelectric coefficients e${}_{ij}$ (in 10${}^{-3}$C/m${}^{2}$ ) of benzil crystals. Experimental data for C${}_{ij}$ amd e${}_{ij}$ are taken from the papers [30,34], respectively.

	Calculation	Experiment
C${}_{11}$	16.66	11.23
C${}_{33}$	12.89	8.56
C${}_{44}$	0.46	1.15
C${}_{66}$	4.67	2.88
C${}_{12}$	7.31	5.47
C${}_{13}$	3.86	3.35
C${}_{14}$	−1.02	−0.61
e${}_{11}$	76.1	120.5
e${}_{14}$	−26.9	−8.0

**Table 2:** Comparison between the phonon frequencies (in cm${}^{-1}$) calculated at the Brillouin zone center (sp. gr. *P3${}_{2}$21*) and the experimental Raman frequencies measured by us in the bulk benzil polycrystals. All experimental frequencies indicated with asterisk (*) were taken from paper [37].

A${}_{1}$		A${}_{2}$	E			
**Calcul.**	**Raman**	**Calcul.**	**Calcul.**	**Raman**	**Calcul.**	**Raman**
27.6	30 *	acoust.	acoust.		992.0	988
41.1	39 *	21.2	11.0	16 *	993.4	1000
73.6	69 *	41.5	39.2	39 *	1009.7	
81.9		66.4	45.6		1011.6	
148.2		79.0	63.8	58 *	1017.9	
158.7	158	134.7	76.9	78 *	1022.8	1021
268.5	271	162.2	79.9		1042.4	1049
318.5		267.9	82.7		1084.7	
394.0	398	393.2	92.9		1088.9	
422.5		420.3	133.3	139	1160.6	
422.5	424	457.3	147.1		1163.2	
609.9		610.5	153.2		1165.5	1167
687.1	687	637.0	161.6	167	1174.7	1178
697.8	702	683.9	264.7		1212.7	1213
721.5	725	715.5	268.4	271	1288.1	
796.5	797	786.6	322.7	336	1304.9	
838.8		841.4	395.8	399	1314.7	
950.8		868.4	399.3		1358.1	1338
978.4		950.4	419.7		1360.2	
993.1	988	975.3	423.4		1442.0	
1010.7		992.5	456.6	462	1445.0	1452
1017.2	1021	1009.4	465.6	473	1476.3	
1042.0	1049	1023.8	610.3		1478.6	1493
1088.7		1086.2	611.3	616	1568.7	
1165.5	1167	1150.8	637.0	644	1569.4	
1170.0		1164.6	681.8		1585.6	1581
1285.7	1292	1211.7	686.7	686	1587.6	
1316.4	1329	1306.9	693.9		1633.0	
1359.4	1351	1359.8	715.7		1642.3	1676
1444.4		1440.5	721.6	725	3107.4	3055
1478.5		1476.1	795.6		3107.5	3065
1569.0		1569.1	797.8	798	3114.7	
1588.4	1595	1589.6	843.8	845	3115.2	
1628.3	1669	1635.0	847.9		3121.6	
3108.0		3107.4	872.5	880	3121.8	
3114.1		3114.6	940.6		3130.5	
3121.6		3122.1	944.1		3130.7	
3130.5		3129.9	979.2		3133.6	3161
3134.6	3161	3133.6	982.2		3134.0	3190

## Supplementary Materials

The following supporting information can be downloaded at: https://www.mdpi.com/article/10.3390/nano13131913/s1. Figure S1. Nitrogen desorption isotherms recorded at *T* = 77 K (a) and extracted the relevant pore size distributions (b) for mesoporous silica membranes of different channel diameters, *D*, see labelled. *R*-scale in section (b) is shifted to the left by the thickness of adsorbed nitrogen monolayer (0.3 nm). The maximum of the distribution curves corresponds to the average channel radius *R${}_{0}$* = *D*/2. *N*/*N${}_{0}$* is the fraction filling, *p*/*ps* is the reduced pressure, *ps* is the bulk saturated pressure of Nitrogen at *T* = 77 K, *R* is the pore radius. Figure S2. Synthesis of silica-benzil nanonocomposites. The dried tubular pSiO${}_{2}$ matrices were subjected to contact with melt benzil (BZL) layer (1) and completely filled with melted benzil by spontaneous capillary inbibition (2) at a temperature a little above the melting point (*T* > *T${}_{m}$* =368 K). A further cooling (2 K/min) of capillary filled free standing membrane (3) down to room temperature leads to the crystallization of the filler and the formation of benzil crystalline nanoclusters inside the silica nanochannels (4). Figure S3. Raman spectrum of bare porous SiO${}_{2}$ membrane, where the peaks at 430, 610, and 800 cm${}^{-1}$ as reported in the literature [45] can be observed. The shape of Raman spectrum of host SiO${}_{2}$ matrix does not depend on pore diameter, 6.0, 7.8, 9.4 or 13.0 nm.
