# Supplementary material for: Nanoarhitectonics of Inorganic–Organic Silica–Benzil Composites: Synthesis, Nanocrystal Morphology and Micro-Raman Analysis
Source: Nanomaterials (Basel). 2023 Jun 23;13(13):1913. doi: 10.3390/nano13131913 (PMC10343406; doi:10.3390/nano13131913)
Supplement: Supplementary file 1 [file nanomaterials-13-01913-s001.zip › nanomaterials-2443968-supplementary.pdf]

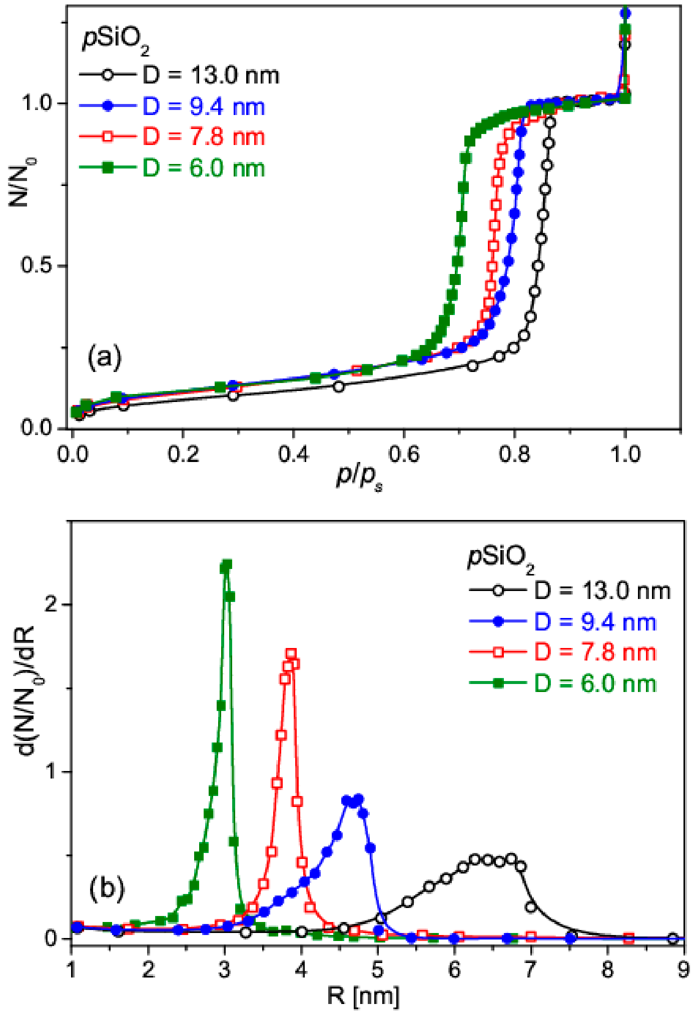

Figure S1. Nitrogen desorption isotherms recorded at T=77K (a) and extracted the relevant pore size distributions (b) for mesoporous silica membranes of different channel diameters, D, see labelled. R-scale in section (b) is shifted to the left by the thickness of adsorbed nitrogen monolayer (0.3 nm). The maximum of the distribution curves corresponds to the average channel radius $R_0=D/2$. N/N_0 is the fraction filling, p/p_s is the reduced pressure, p_s is the bulk saturated pressure of Nitrogen at T=77K, R is the pore radius.

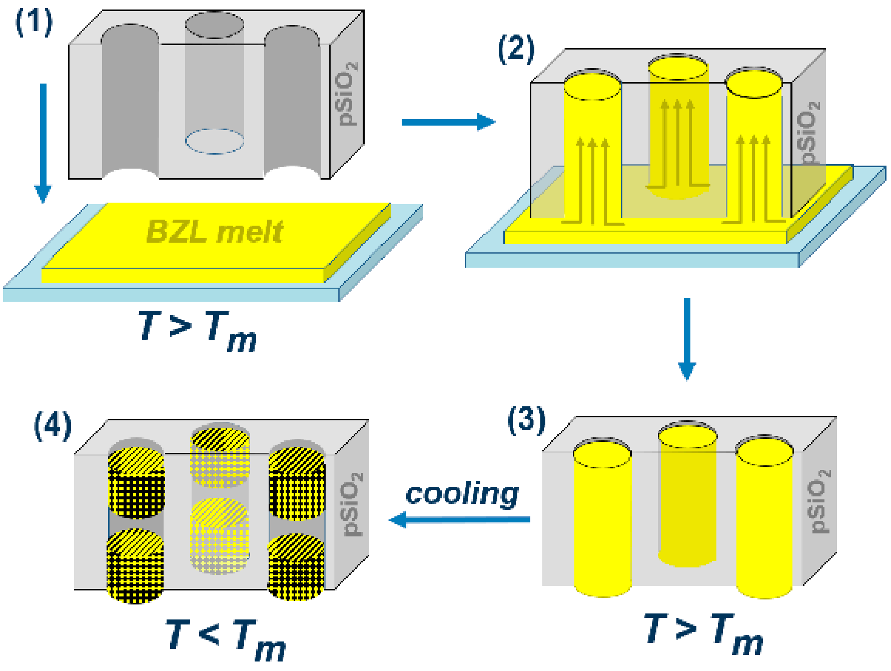

Figure S2. Synthesis of silica-benzil nanonocomposites. The dried tubular pSiO2 matrices were subjected to contact with melt benzil (BZL) layer (1) and completely filled with melted benzil by spontaneous capillary inbibition (2) at a temperature a little above the melting point ($T > T_m$ =368 K). A further cooling (2K/min) of capillary filled free standing membrane (3) down to room temperature leads to the crystallization of the filler and the formation of benzil crystalline nanoclusters inside the silica nanochannels (4).

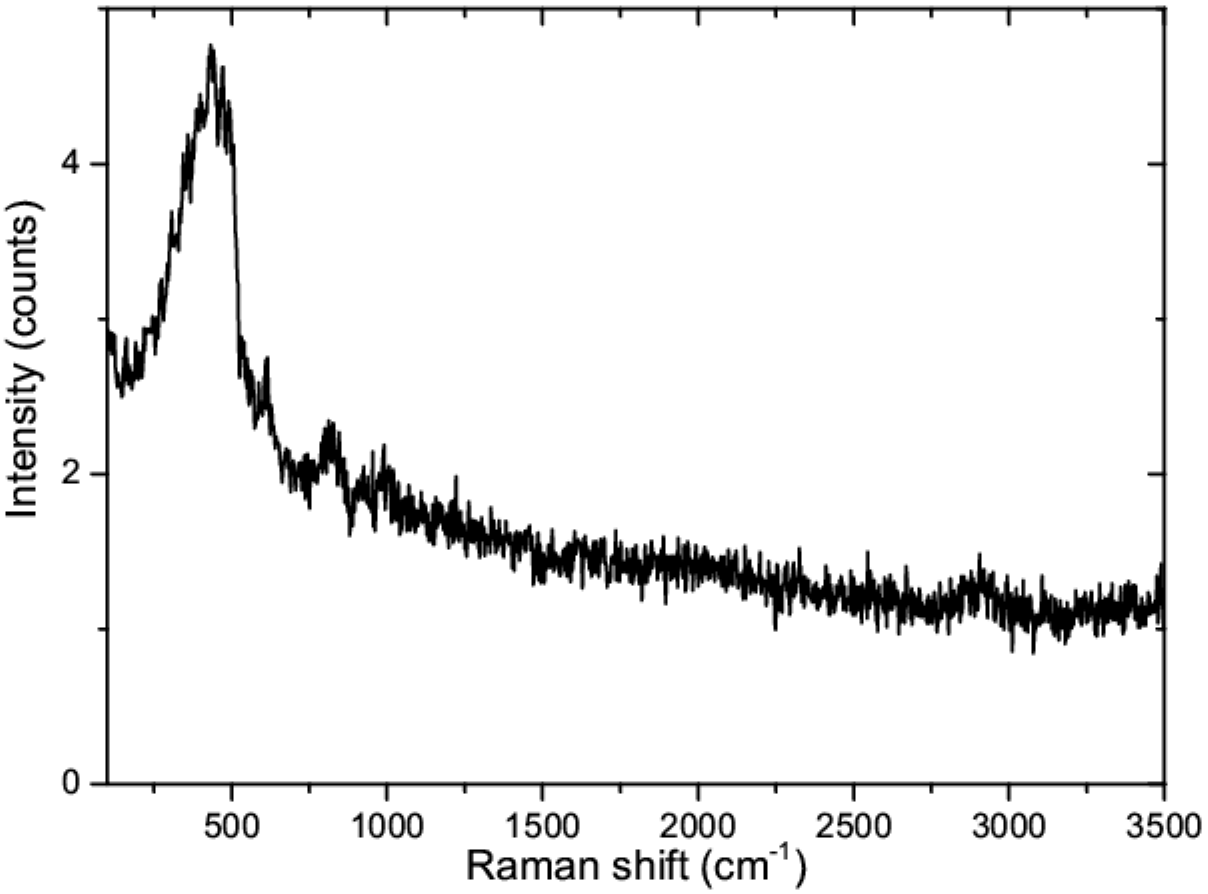

Figure S3. Raman spectrum of bare porous SiO_2 membrane, where the peaks at 430, 610, and 800 cm^{-1} as reported in the literature [S1] can be observed. The shape of Raman spectrum does not depend on pore diameter, 6.0, 7.8, 9.4 or 13.0 nm.

[S1]. F. L. Galeener and A. E. Geissberger, Vibrational dynamics in ^{30}Si-substituted vitreous SiO_2. *Phys. Rev. B*, 27:6199-6204, 1983
